# Assessment of genetic diversity in Coho salmon (*Oncorhynchus kisutch*) populations with no family records using ddRAD-seq

**DOI:** 10.1186/s13104-018-3663-4

**Published:** 2018-08-02

**Authors:** Sho Hosoya, Kiyoshi Kikuchi, Hiroshi Nagashima, Junichi Onodera, Kouichi Sugimoto, Kou Satoh, Keisuke Matsuzaki, Masaki Yasugi, Atsushi J. Nagano, Akira Kumagayi, Kenichi Ueda, Tadahide Kurokawa

**Affiliations:** 1 Fisheries Laboratory, Graduate School of Agricultural and Life Sciences, University of Tokyo, 2971-4 Bentenjima, Maisaka, Hamamatsu, Shizuoka 431-0214 Japan; 2 Miyagi Prefecture Fisheries Technology Institute, Freshwater Fisheries Experimental Station, Taiwa, Miyagi 981-3625 Japan; 3 Center for Ecological Research, Kyoto University, Hirano 509-3-2, Otsu, Shiga 520-2113 Japan; 4 JST CREST, Honcho 4-1-8, Kawaguchi, Saitama 332-0012 Japan; 5 Faculty of Agriculture, Ryukoku University, Yokotani 1-5, Seta Ohe-cho, Otsu-shi, Shiga 520-2194 Japan; 6 Kushiro Laboratory, Hokkaido National Fisheries Research Institute, Japan Fisheries Research and Education Agency, 116 Katsurakoi, Kushiro-shi, Hokkaido 085-0802 Japan

**Keywords:** Breeding value, Coho salmon (*Oncorhynchus kisutch*), ddRAD-seq, Genomic best linear unbiased prediction, Genetic diversity, Heritability, Prediction accuracy, Selective breeding, SNPs

## Abstract

**Objective:**

Selective breeding for desirable traits is becoming popular in aquaculture. In Miyagi prefecture, Japan, a selectively bred population of Coho salmon (*Oncorhynchus kisutch*) has been established with the original, randomly breeding population maintained separately. Since they have been bred without family records, the genetic diversity within these populations remains unknown. In this study, we estimated the genetic diversity and key quantitative genetic parameters such as heritability and genomic breeding value for body size traits by means of genomic best linear unbiased prediction to assess the genetic health of these populations.

**Results:**

Ninety-nine and 83 females from the selective and random groups, respectively, were genotyped at 2350 putative SNPs by means of double digest restriction associated DNA sequencing. The genetic diversity in the selectively bred group was low, as were the estimated heritability and prediction accuracy for length and weight (*h*^2^ = 0.26–0.28; accuracy = 0.34), compared to the randomly bred group (*h*^2^ = 0.50–0.60; accuracy = 0.51–0.54). Although the tested sample size was small, these results suggest that further selection is difficult for the selectively bred population, while there is some potential for the randomly bred group, especially with the aid of genomic information.

**Electronic supplementary material:**

The online version of this article (10.1186/s13104-018-3663-4) contains supplementary material, which is available to authorized users.

## Introduction

The aquaculture production of Coho salmon (*Oncorhynchus kisutch*) in Japan started in the late 1970s using populations imported from North America. Since then, the species is important in Japanese fish production as ranked fourth among marine aquaculture fish in 2014, with a harvest of 12,800 tonnes [1]. This quantity can be increased significantly with selective breeding for growth rate. For this purpose, a selection program for body size was begun at Miyagi Prefectural Fisheries Research Station starting with 16 selected females and 13 randomly chosen males, with the original, randomly breeding population maintained separately. However, since there was no pedigree, the extent of genetic relatedness among the individuals was unknown for both populations.

The future of the ongoing breeding programs depends on the existing genetic diversity in the given population [2]. Thus, it was necessary to assess the extent of genetic diversity to maintain the health of the populations, rendering the maintenance of accurate family records essential. Recent advances in genome-wide single nucleotide polymorphisms (SNPs) genotyping permit a fine-grain assessment of the current level of genetic diversity, even for the population without family records.

In this study, we genotyped genome-wide SNPs collected by means of double digest restriction associated DNA sequencing (ddRAD-seq) [3] for the selectively bred (SB) and the randomly breeding (RB) populations to infer the genetic relatedness between individuals within each population. We then estimated the heritability and genomic breeding values for body weight (BW) and fork length (FL) at 47 months post fertilization to examine the possibility of selective breeding using genomic information (genomic selection) of these populations.

## Main text

### Methods

#### Samples

Both populations (SB and RB) are maintained at the Inland Fisheries Experimental Station, Miyagi Prefecture Fisheries Technology Center (Miyagi, Japan), with a largely unknown family history. The original population was introduced from Lower Kalama hatchery (WA, USA) to Japan in 1978. This population was maintained without individual or family identification until 2000 when the first phenotypic selection was done using 29 individuals, followed by a second selection in 2003 using 50 individuals. The population was then bred randomly two times, once in 2006 (198 individuals) and again in 2009 (94 individuals). The progeny produced in 2009 were used for the subsequent genetic and phenotypic analyses in this study. The RB population used in this study was also produced in 2009 by random crosses among individuals from the original population. The two populations were reared separately throughout the experiment. At 47 months post fertilization, 1181 and 558 individuals were sampled from SB and RB, respectively, and the fork length and weight were measured. “Jack” males, which mature at a very early age [4], and other males that also matured somewhat early (3 years) were excluded from the populations, potentially distorting the genetic diversity among the males. Therefore, we used only females in this study (*n* = 100/population).

#### Genotyping

Genomic DNA was extracted from the caudal fin using the FUJIFILM QuickGene-810 extraction platform (Fujifilm, Japan) following the manufacturer’s instructions. ddRAD-seq was done following Sakaguchi et al. [5]. *Bgl*II and *Eco*RI were used for genomic DNA digestion. Sequencing of 100 bp paired-end reads and the index sequence of the library was done using HiSeq2500 (Illumina) with TruSeq v3 chemistry on two lanes. Reads were trimmed using Trimmomatic-0.35 [6] with the following parameters: ILLUMINACLIP TruSeq3-PE-2.fa:2:30:10, LEADING:19, TRAILING:19, SLIDINGWINDOW:30:20, AVGQUAL:20, and MINLEN:101. After filtering, an average approximately 2 million reads per individual were obtained. Samples with less than 60,000 reads (17 samples from RB and one from SB) were excluded. The remaining reads at both ends were mapped to the Coho salmon reference genome (Okis_V1; GenBank assembly accession: GCA_002021735.1) using BWA-mem [7] with default settings. Reads of mapping quality (MAPQ) less than 4 were removed. SNP calling was done using Stacks (ver 1.45) [8]. All the ref_map.pl parameters were set to default except for the following: minimum depth of coverage (-m = 5). We set minimum depth of coverage to 5 following Dodd et al. [9] who suggested that the minimal sequencing depth is around 2–4 for relatedness between individuals and 5–10 for self-relatedness. The rxstacks program was applied for genotype calling in individual samples using log likelihood filtering (–lnl_lim = − 120) followed by the cstacks and sstacks programs, which yielded a total of 378,125 loci. After the RAD loci with more than 3 SNPs and 3 alleles were filtered out, 43028 RAD loci remained. The RAD loci were selected under following criteria: (1) SNPs that genotyped more than 50% of the individuals, and those that genotyped more than 90% of the individuals, for both families and (2) minor allele frequency (MAF) was larger than 0.05. For the RAD loci with two SNPs, one of the SNPs was randomly selected by Stacks population program. With the filtration threshold of MAPQ (≥ 4), MAF (≥ 0.05) and number of alleles (= 2), it is expected that most of SNPs from paralogs regions were removed. We did not filtered out SNPs not in the Hardy–Weinberg equilibrium, because such SNPs are expected in the selected population with small effective population size and not necessarily removed [8]. Finally, 2350 (50% genotyped) and 1064 (90% genotyped) putative SNP loci remained. These SNP sets are referred to as 1K-SNPs and 2K-SNPs, respectively. Missing genotype data of 1K- and 2K-SNPs were imputed using Beagle (v4.1) [10]. The genetic analyses were done using 1-K SNPs and estimation of heritability and GEBV were done using 2K-SNPs.

#### Genetic analysis

Kin relationships among individuals were inferred using KING [11]. First, second and third degree relationships within pairs were determined using kinship coefficient ranges of > 0.177, 0.0884–0.177 and 0.0442–0.0884, respectively [11]. We also estimated effective population size using the Linkage Disequilibrium method implemented in NeEstimatorV2.1 (the lowest allele frequency = 0.05) [12].

Heritability estimation and genomic prediction of FL and BW were done for SB and RB by means of genomic best linear unbiased prediction (GBLUP) implemented in the R package, rrBLUP [13]. The REML (restricted maximum likelihood) estimates of the variance components and BLUP solution for genomic breeding values (GEBV) were obtained using the kin.blup function. The narrow sense heritability was calculated as *h*^*2*^ = *σ*_a_^2^/*σ*_p_^2^, where *σ*_a_^2^ is the additive genetic variance and *σ*_p_^2^ is the total phenotypic variance. The prediction accuracy of GEBV was calculated using a fivefold cross validation design following Tsai et al. [14] with some modifications; the cross validation procedure was repeated ten times independently to obtain the mean and the standard error of the measure of accuracy. At first, each population was randomly divided into five subsets, one for validation and the remaining for training. The phenotypes of the validation set were masked and GEBV of these individuals were estimated from the training set using the kin.blup function of rrBLUP. This step was repeated five times in total while rotating the validation sets. Accuracy was calculated as the average of the correlation between the GEBV and the observed phenotypes of the validation set divided by the square root of the heritability estimated from all individuals. The whole procedure was repeated ten times independently to calculate the mean and the standard error of the measure of accuracy.

### Results

It was confirmed by *t*-test that SB (*n* = 99) was significantly larger than RB (*n* = 83) in FL (*P* = 0.003) and BW (*P* = 0.000014). Estimation of traditional pedigree-based relatedness was not possible for either population since the family history had not been recorded. However, our genome-wide SNP data enabled us to infer the kin relationship among the individuals. These results revealed the genetic relatedness among the individuals of the selected (SB) population; 33.9% of the individual pairs had at least a third degree relationship (compared to 23.6% in the randomly breeding (RB) population) (Table 1, Additional file 1: Fig. S1). Reflecting the close genetic relatedness, the estimated effective population size for SB (*Ne* = 36.9) was smaller than for RB (*Ne* = 43.8) (Table 2).

**Table 1:** Percentage of individual pairs in kin relationships within and between populations

	1st degree (%)	2nd degree (%)	3rd degree (%)	Total pairs
Between populations	0.0	0.0	0.0	8217
Randomly bred (RB)	0.7	8.8	14.1	3403
Selectively bred (SB)	1.2	14.4	18.3	4851

**Table 2:** Effective population size estimated by means of linkage disequilibrium method

	*Ne*	95% confidence interval	
		Lower	Upper
Randomly bred (RB)	43.8	43.4	44.3
Selectively bred (SB)	36.9	36.5	37.2

Heritability and prediction accuracy for FL and BW were estimated using 2-K SNPs (Table 3). For both of the traits, a drop in heritability was observed in SB (*h*^*2*^ = 0.26–0.28) compared to RB (*h*^*2*^ = 0.50–0.60). Similarly, the prediction accuracies were low for SB (accuracy = 0.33–0.34), while those for RB were relatively high (accuracy = 0.51–0.59), although a strong correlation between the predicted and the observed phenotypes was seen for both traits in both the populations.

**Table 3:** Mean body size, additive and residual variations, heritability and prediction accuracy (mean ± SE) for body size traits (FL and BW) of the two populations

Traits	Stats	Randomly bred (RB)	Selectively bred (SB)
FL	Mean ± SD (cm)	40.6 ± 2.6	42.0 ± 3.7
	Heritability	0.60	0.28
	Correlation^a^	0.97	0.89
	Accuracy	0.54 ± 0.02	0.34 ± 0.04
BW	Mean ± SD (g)	858 ± 176	1003 ± 259
	Heritability	0.50	0.26
	Correlation^a^	0.95	0.88
	Accuracy	0.51 ± 0.03	0.34 ± 0.05

^a^Between the observed and the predicted phenotypes

### Discussion

The 1K-SNPs data obtained by means of ddRAD-seq enabled us to infer kin relationship among individuals. High degree of genetic relatedness and decreased effective population size were clearly observed in the selectively bred (SB) population when compared to the original, randomly bred, (RB) population (Tables 1, 2). The small population size and the high genetic relatedness evidently resulted in reduced additive genetic variance (*σ*_a_^2^) and therefore, heritability (Table 3), both of which can indicate excessive inbreeding [15]. The differences in heritability between the two populations seemed larger than in *Ne*. This will be partly because additive genetic variation was substantially reduced in SB as selection and inbreeding decreases heritabilities for polygenic traits including body size [15, 16], while the two rounds of random mating might increase *Ne* without increasing additive genetic variance in SB population. Low values of predictability in SB could also be the consequence of exhaustion of genetic diversity within a few generations because SB was established from a limited broodstock on the one hand, and with a high degree of genetic relatedness on the other. All those results suggest the difficulty of continuation of breeding program for this population without restoration of genetic diversity by introduction of new genetic material from other populations.

In contrast, the genetic diversity in RB seemed to be high enough for a breeding value prediction and genomic selection for body length and weight, since estimated heritability and prediction accuracy were relatively high (*h*^*2*^ = 0.50–0.60; accuracy = 0.51–0.59). The estimated effective population size (*Ne* = 43.8), however, suggests genetic diversity will be exhausted within several rounds of selection. One possible approach to apply selective breeding for these populations is to use genomic selection to select individuals from RB for crossing with individuals from SB. This will permit some restoration of the genetic diversity in SB with the minimum loss in its growth performance, and maintenance of the breeding program in SB, simultaneously.

## Limitation

Our results demonstrate that ddRAD-seq worked well for the assessment of the current level of genetic diversity of the two Coho salmon populations bred without family records. High prediction accuracies for fork length and weight were observed in the randomly breeding population. However, it should be noted that some of the difference between populations could be due to tank effects since each population was raised in a single tank. Moreover, these analyses were done with the limited numbers of samples and SNPs, and thus, the estimated statistics are expected to have high variation. Therefore, the success and failure of the genomic selection for these populations should also be tested using large sample/SNP sizes.

## Additional file

**Additional file 1: Fig. S1.** Kin relationships among individuals. Small rectangles on the outer edge refer to individual fish. Pairs of individuals with first and second degree relationship are connected with dark and light lines, respectively.

## Abbreviations

SB: selectively bred population
RB: randomly bred population
SNPs: single nucleotide polymorphisms
ddRAD-seq: double digest restriction associated DNA sequencing
BW: body weight
FL: fork length
GBLUP: genomic best linear unbiased prediction
GEBV: genomic breeding value
REML: restricted maximum likelihood
