# Supplementary material for: Assessment of genetic diversity in Coho salmon (Oncorhynchus kisutch) populations with no family records using ddRAD-seq
Source: BMC Res Notes. 2018 Aug 2;11:548. doi: 10.1186/s13104-018-3663-4 (PMC6071332; doi:10.1186/s13104-018-3663-4)
Supplement: Supplementary file 1 — Additional file 1: Fig. S1. Kin relationships among individuals. Small rectangles on the outer edge refer to individual fish. Pairs of individuals with first and second degree relationship are connected with dark and light lines, respectively. [file 13104_2018_3663_MOESM1_ESM.pdf]

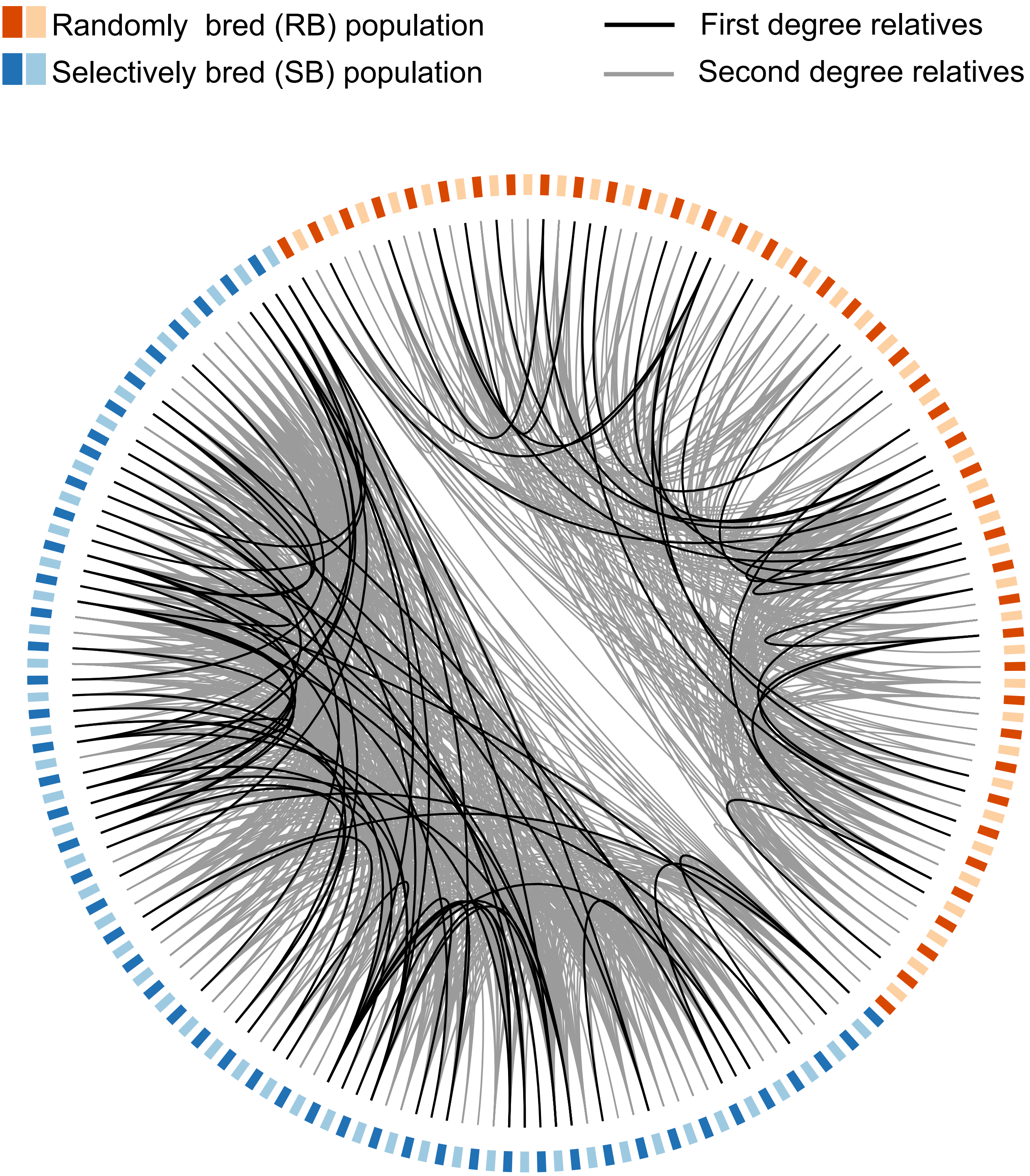

Additional file1: Fig. S1 Kin relationships among individuals. Small rectangles on the outer edge refer to individual fish. Pairs of individuals with first and second degree relationship are connected with dark and light lines, respectively.
